# Supplementary material for: Polyketide synthase 12 is an in vivo essential source of novel mycolyl lipids in Mycobacterium tuberculosis
Source: bioRxiv. 2026 Sep 25:2026.09.25.754250. Preprint. [Version 1] doi: 10.64898/2026.09.25.754250 (PMC13622277; doi:10.64898/2026.09.25.754250)
Supplement: Supplementary file 1 [file NIHPP2026.09.25.754250v1-supplement-4.pdf]

bioRxiv preprint doi: https://doi.org/10.64898/2026.09.25.754250; this version posted September 25, 2026. The copyright holder for this preprint (which was not certified by peer review) is the author/funder, who has granted bioRxiv a license to display the preprint in perpetuity. It is made available under a CC-BY 4.0 International license.

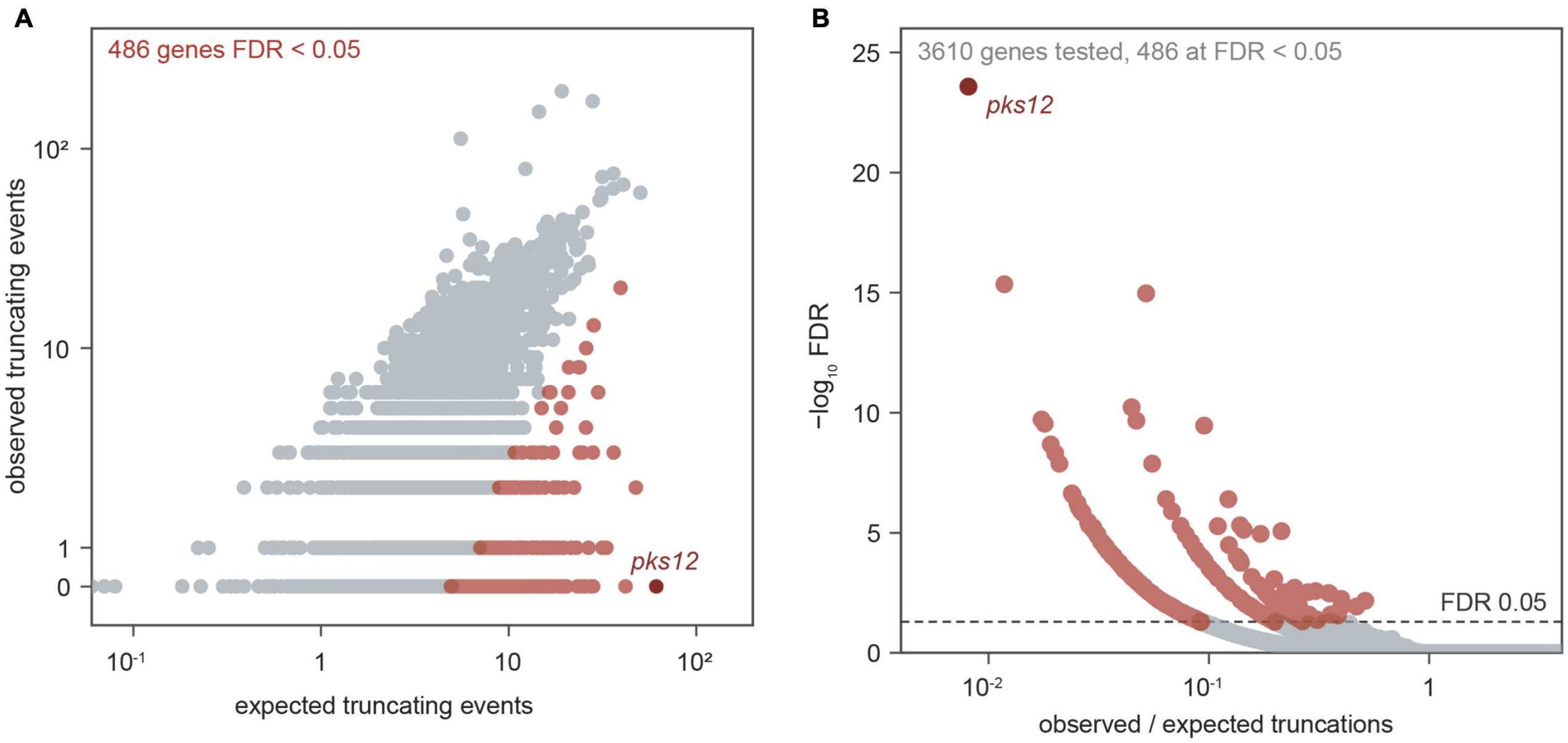

Fig. S1. *pks12* is the most significantly under-truncated gene in Mtb. (**A**) Anticipated versus measured gene truncations via premature stop codon mutation, for all mappable genes in the Mtb genome, with expectations set by gene-specific mutational opportunity and measured genome-wide mutation rates. (**B**) Significance of lower-than-expected truncation rates as measured by false discovery rate (FDR) following Benjamini-Hochberg correction.

bioRxiv preprint doi: https://doi.org/10.64898/2026.09.25.754250; this version posted September 25, 2026. The copyright holder for this preprint (which was not certified by peer review) is the author/funder, who has granted bioRxiv a license to display the preprint in perpetuity. It is made available under a CC-BY 4.0 International license.

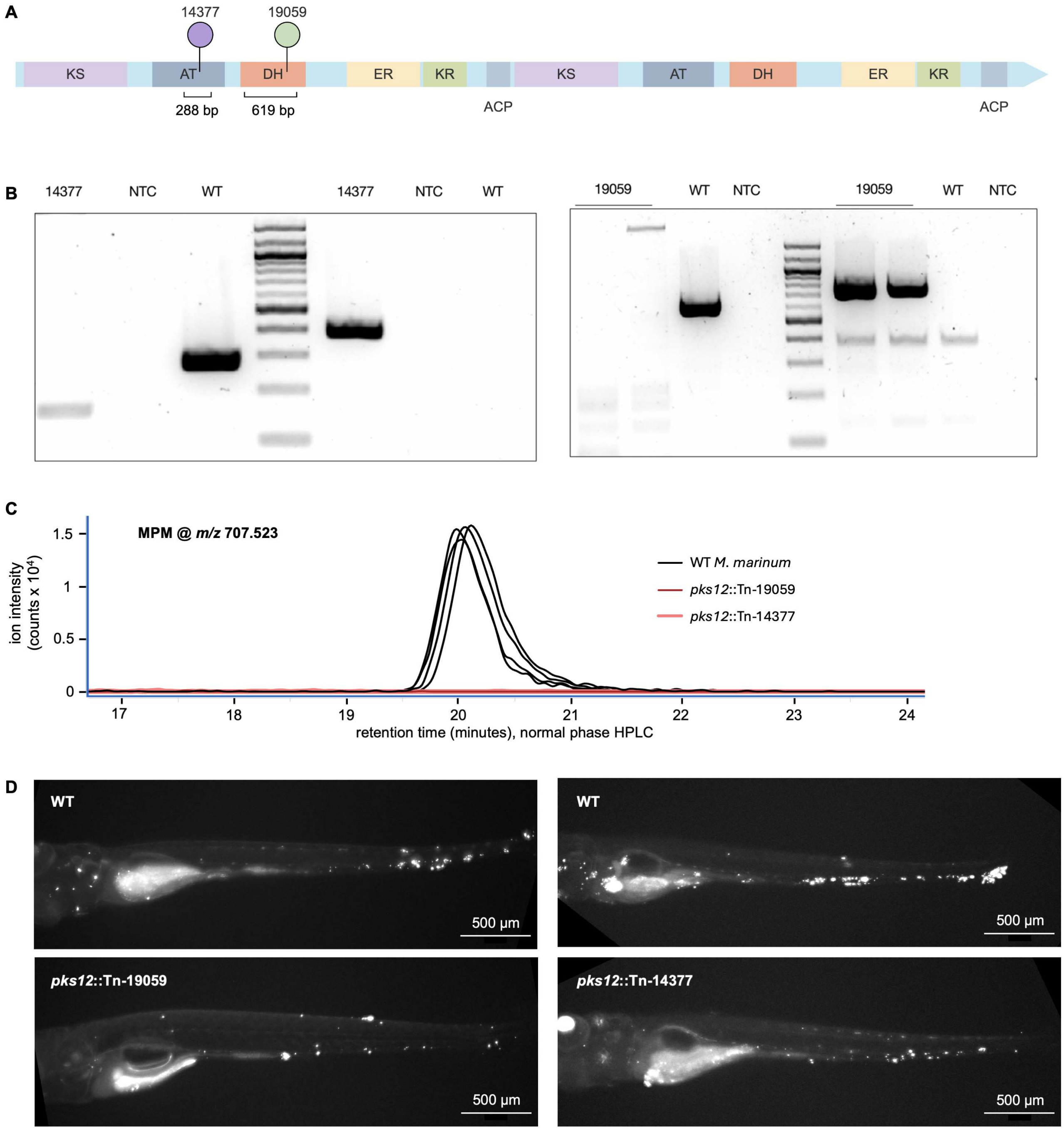

Fig. S2. Analysis of *pks12* transposon mutants in *M. marinum*. (**A**) Schematic representation of the *M. marinum* MMAR_3025/*pks12* coding sequence, showing predicted protein domains and the positions of two independent transposon insertions, *pks12*::Tn-14377 and *pks12*::Tn-19059. Domains are labeled as ketoacyl synthase (KS), acyltransferase (AT), dehydratase (DH), enoyl reductase (ER), ketoreductase (KR), and acyl carrier protein (ACP). The schematic was generated using the IBS 2.0 web server [ref]. (**B**) PCR analysis of *pks12*::Tn-14377 and *pks12*::Tn-19059 compared with WT, no-template control (NTC), and TriDye 100 bp DNA Ladder (New England Biolabs N3271S). WT PCR amplicon sizes ran as expected compared to a DNA ladder: 288 bp for the *pks12*::Tn-14377 locus and 619 bp for the *pks12*::Tn-19059 locus. Mutant amplicons are expected to be approximately 100 bp larger because of the transposon insertion. (**C**) High performance liquid chromatography-mass spectrometry (HPLC-MS) chromatogram for the Pks12 lipid product mannosyl phosphomycoketide (MPM) in WT and *pks12*::Tn strains. (**D**) Representative fluorescence images at 5 days post-infection of zebrafish larvae infected with an initial dose of 150–250 fluorescent bacilli, shown for *pks12*::Tn-14377 and *pks12*::Tn-19059 mutants and intra-experimental matched WT animals.

bioRxiv preprint doi: https://doi.org/10.64898/2026.09.25.754250; this version posted September 25, 2026. The copyright holder for this preprint (which was not certified by peer review) is the author/funder, who has granted bioRxiv a license to display the preprint in perpetuity. It is made available under a CC-BY 4.0 International license.

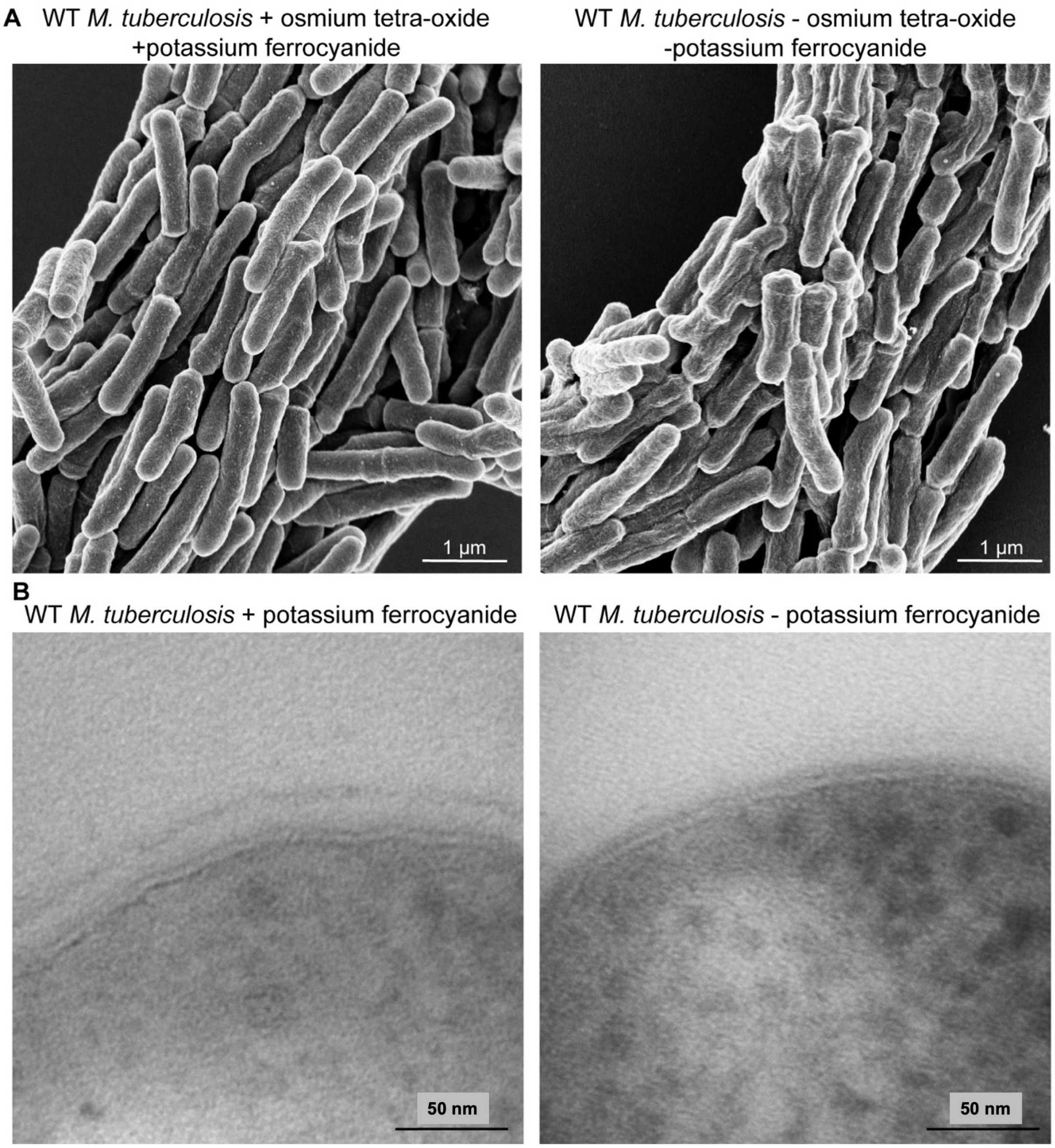

Fig. S3. Potassium ferrocyanide improves Mtb structure in SEM and shows the capsular layer in TEM. (**A**) Representative SEM images of WT Mtb with and without osmium tetra-oxide (osmium tetroxide), showing preservation of intact surface morphology. (**B**) Representative TEM images of WT Mtb embedded with or without potassium ferrocyanide, showing preservation of the capsular glycan layer.

bioRxiv preprint doi: https://doi.org/10.64898/2026.09.25.754250; this version posted September 25, 2026. The copyright holder for this preprint (which was not certified by peer review) is the author/funder, who has granted bioRxiv a license to display the preprint in perpetuity. It is made available under a CC-BY 4.0 International license.

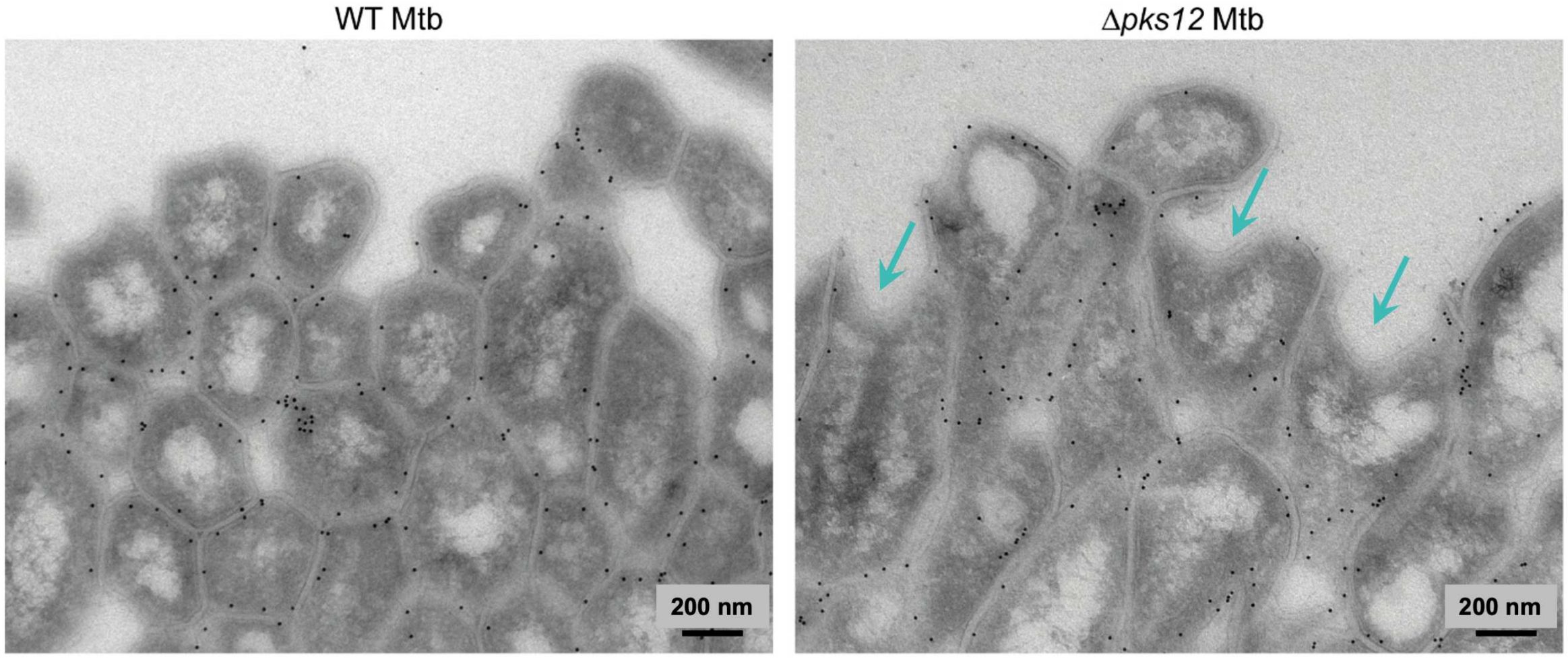

Fig. S4. Indentations in Δ*pks12* Mtb extend to all layers of the cell envelope. Representative TEM images of cryo-sections of *M. tuberculosis* cords of WT Mtb (left) and Δ*pks12* Mtb (right). Cyan arrows indicate bacterial indentations. Sections were labelled with CS-35 antibody immunogold.

bioRxiv preprint doi: https://doi.org/10.64898/2026.09.25.754250; this version posted September 25, 2026. The copyright holder for this preprint (which was not certified by peer review) is the author/funder, who has granted bioRxiv a license to display the preprint in perpetuity. It is made available under a CC-BY 4.0 International license.

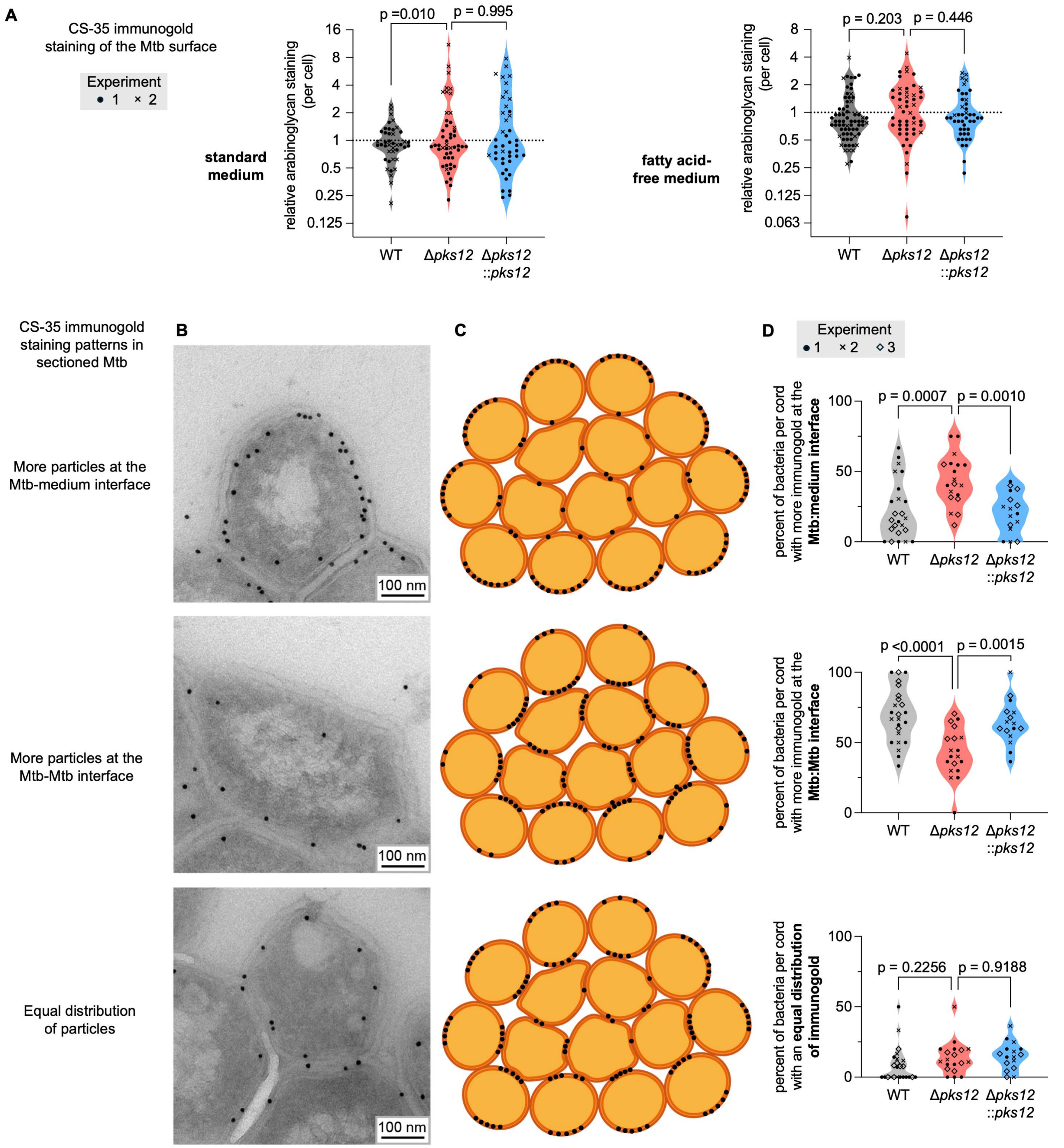

Fig. S5. Effects of deleting *pks12* on arabinoglycan surface exposure and distribution. (**A**) Quantification of of CS-35 immunogold staining for surface arabinoglycans on intact WT, Δ*pks12,* and complemented Mtb, grown in different media. Data points represent individual bacteria across two independent experiments, indicated by different symbols; statistics were performed using linear mixed-effects models with Tukey's multiple comparisons test. (**B**) Representative TEM images of bacteria with differential localization of CS-35 immunogold between the Mtb-Mtb cellular interface and the Mtb-medium interface. (**C**) Schematic representation of Mtb within a cord (cross-sectioned) with differential localization of CS-35 immunogold, as described in (B); orange represents the cytosol and dark orange the cell envelope, while black dots represent CS-35 immunogold. (**D**) Quantification of the percentage of Mtb per cord with with differential localization of CS-35 immunogold. Data points represent cords from 3 individual experiments, indicated by different symbols; statistics were performed using an ordinary one-way ANOVA with Dunnett's multiple comparisons test.

bioRxiv preprint doi: https://doi.org/10.64898/2026.09.25.754250; this version posted September 25, 2026. The copyright holder for this preprint (which was not certified by peer review) is the author/funder, who has granted bioRxiv a license to display the preprint in perpetuity. It is made available under a CC-BY 4.0 International license.

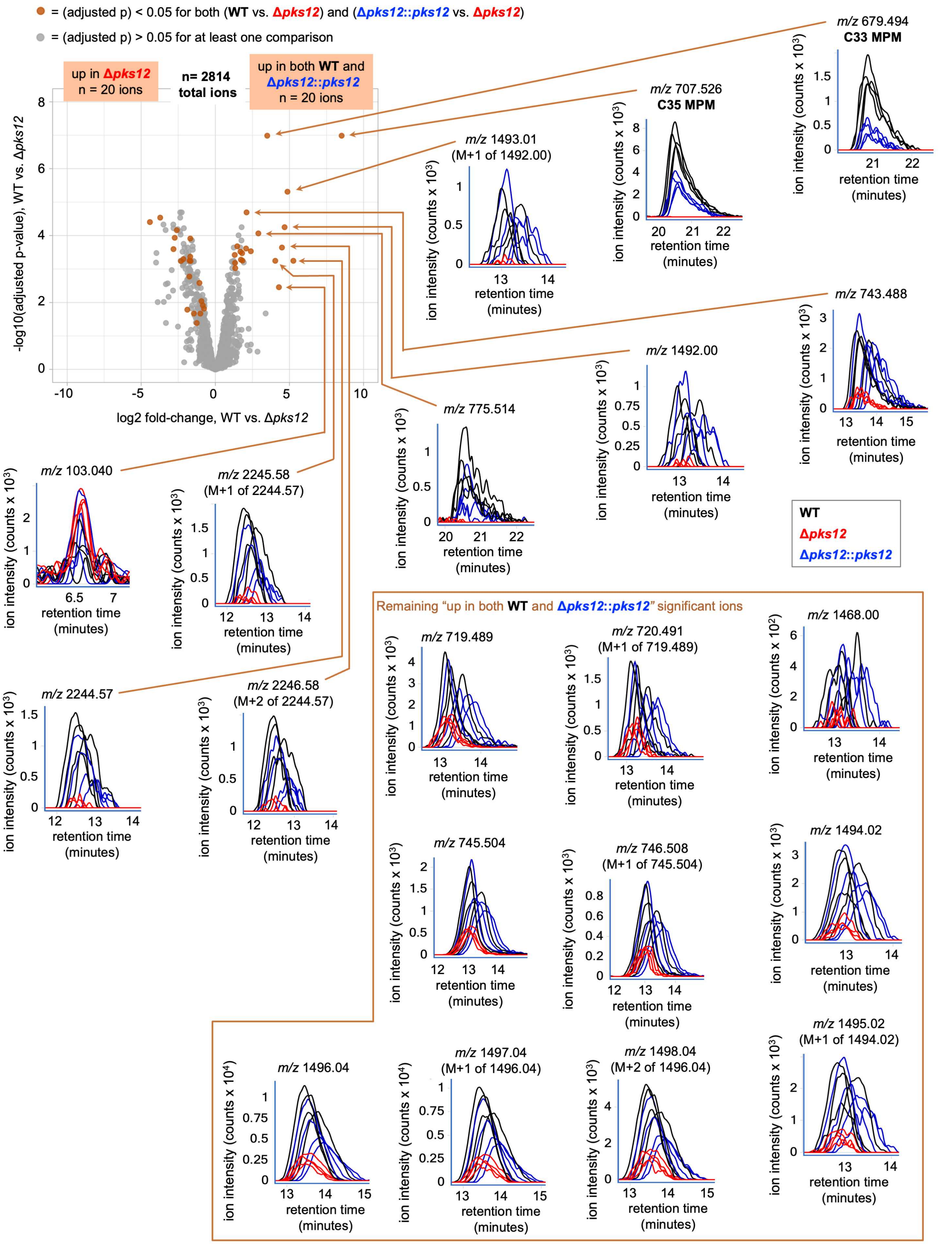

Fig. S6. Lipidomics with automated peak picking does not identify any non-MPM *pks12* products. Lipidomic comparison of WT and complemented (Δ*pks12*::*pks12*) Mtb with Δ*pks12* Mtb; volcano plot for WT vs. Δ*pks12* shown (top left). Chromatograms are shown for all 20 ions that are significantly lower (adjusted $p < 0.05$) in the Δ*pks12* mutant for both comparisons (WT vs. Δ*pks12* and Δ*pks12*::*pks12* vs. Δ*pks12*). Ions are labeled with XCMS-calculated m/z values; it is noted when they are derived from known lipids or when they are ^{13}C isotopes (M+n) of other significant ions. Only the two MPM ions with completely flat Δ*pks12* (red) chromatograms are unambiguously and exclusively produced downstream of *pks12*.

bioRxiv preprint doi: https://doi.org/10.64898/2026.09.25.754250; this version posted September 25, 2026. The copyright holder for this preprint (which was not certified by peer review) is the author/funder, who has granted bioRxiv a license to display the preprint in perpetuity. It is made available under a CC-BY 4.0 International license.

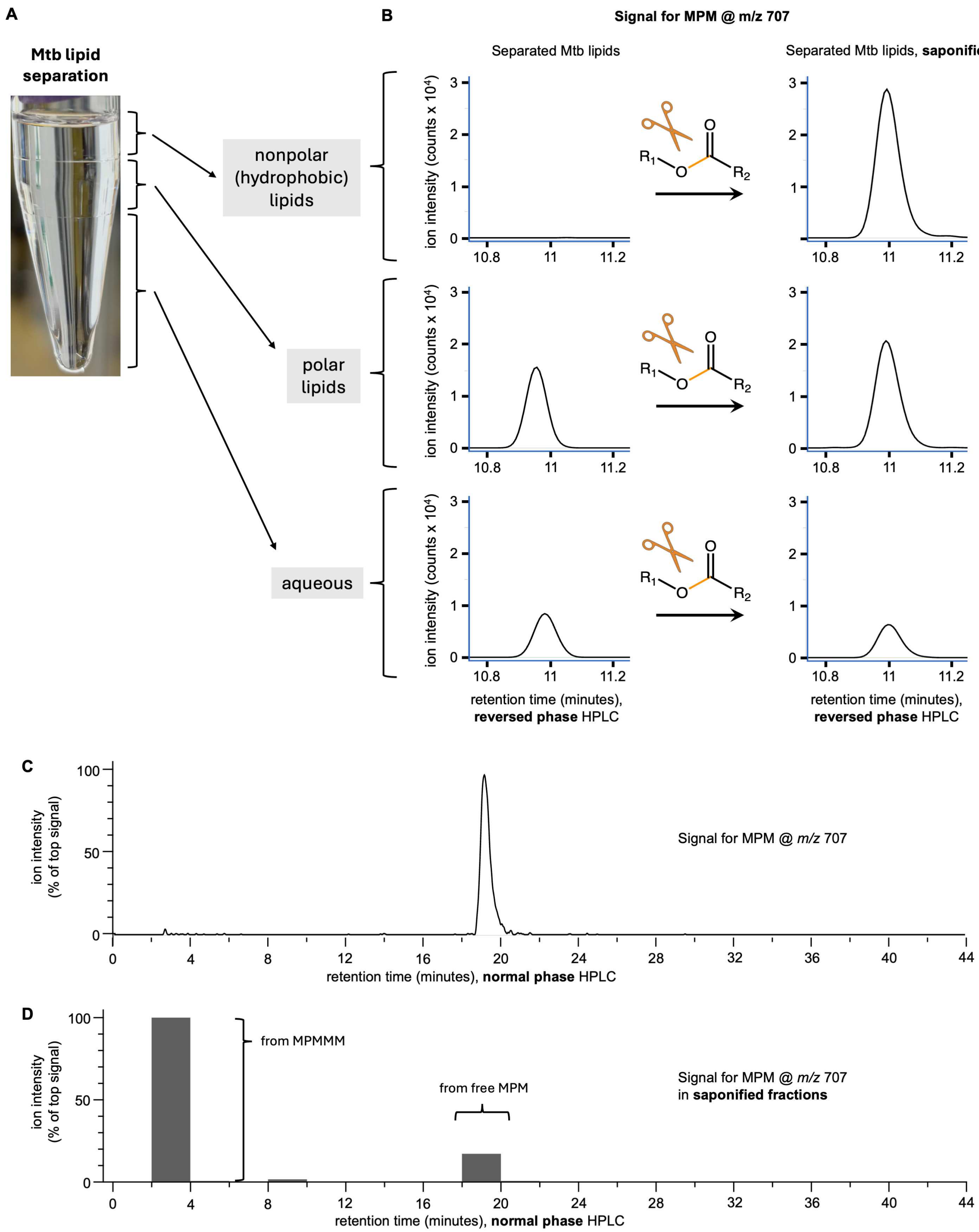

Fig. S7. Saponifiable MPM is released from hydrophobic material. (**A**) 3-phase method used to separate Mtb lipids. (**B**) Targeted analysis of the 3 separated phases of Mtb lipids for MPM (by reversed phase HPLC-ESI-MS) before (left) and after saponification (right). (**C**) Normal phase HPLC-ESI-MS analysis of Mtb total lipids, with targeted analysis for MPM. (**D**) Fractionation of Mtb total lipids by normal phase HPLC with collection every two minutes, followed by saponification, and then targeted analysis for MPM (by reversed phase HPLC-ESI-MS). Data shown are representative of three independent experiments.

bioRxiv preprint doi: https://doi.org/10.64898/2026.09.25.754250; this version posted September 25, 2026. The copyright holder for this preprint (which was not certified by peer review) is the author/funder, who has granted bioRxiv a license to display the preprint in perpetuity. It is made available under a CC-BY 4.0 International license.

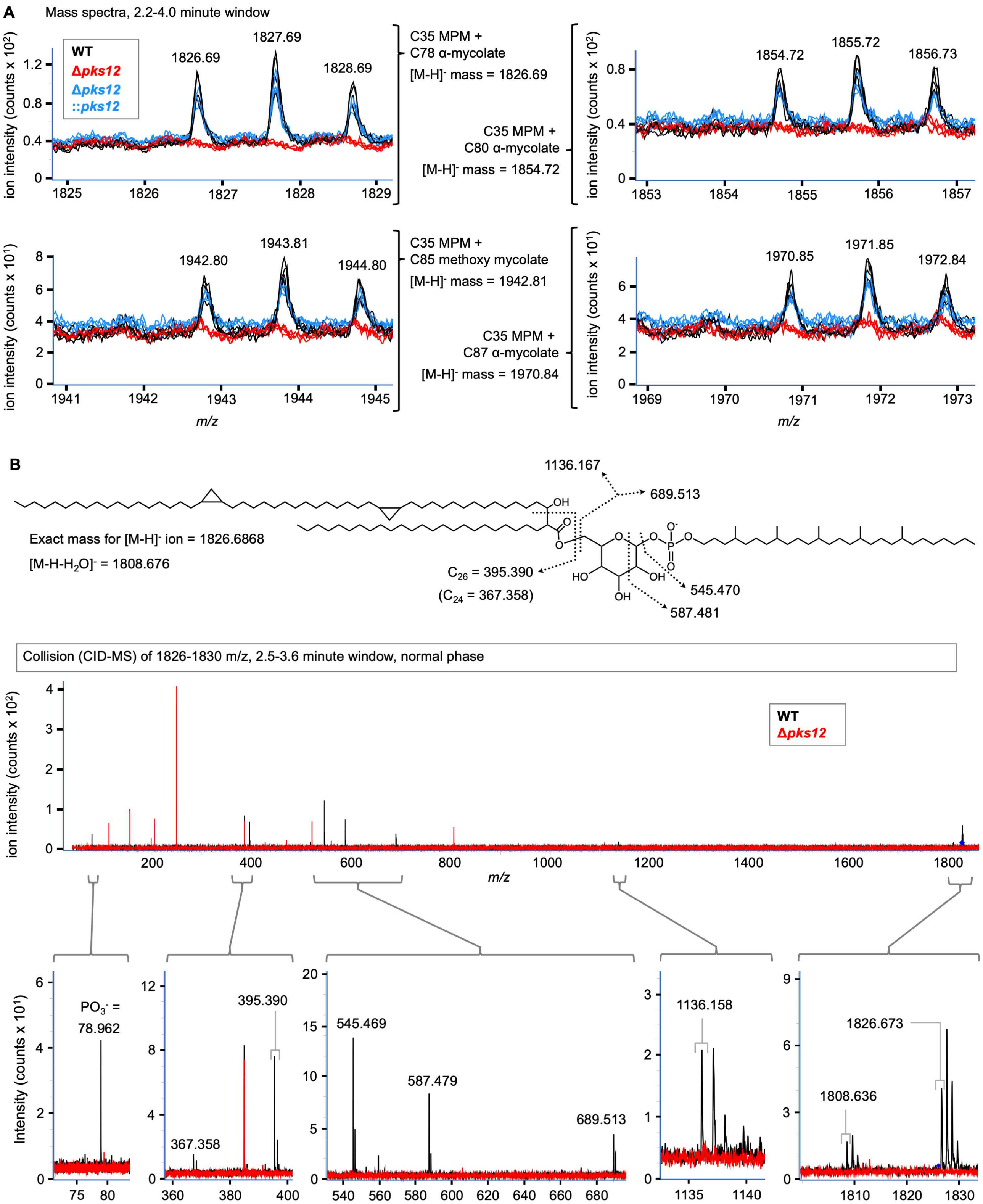

Fig. S8. A mycolated form of MPM, MPMMM, is *pks12*-dependent. (**A**) HPLC-ESI-MS spectra for four MPMMM molecules with differing mycolyl chain length. (**B**) Collisional mass spectrometry (CID-MS) for the most abundant form of MPM at *m/z* 1826 (including abundant ^{13}C isotopes), with Δ*pks12* CID-MS products used to filter out nonspecific (red) fragment ion peaks. All peak *m/z* values were assigned by MassHunter software, all ion mass values were calculated from chemical formulae.

bioRxiv preprint doi: https://doi.org/10.64898/2026.09.25.754250; this version posted September 25, 2026. The copyright holder for this preprint (which was not certified by peer review) is the author/funder, who has granted bioRxiv a license to display the preprint in perpetuity. It is made available under a CC-BY 4.0 International license.

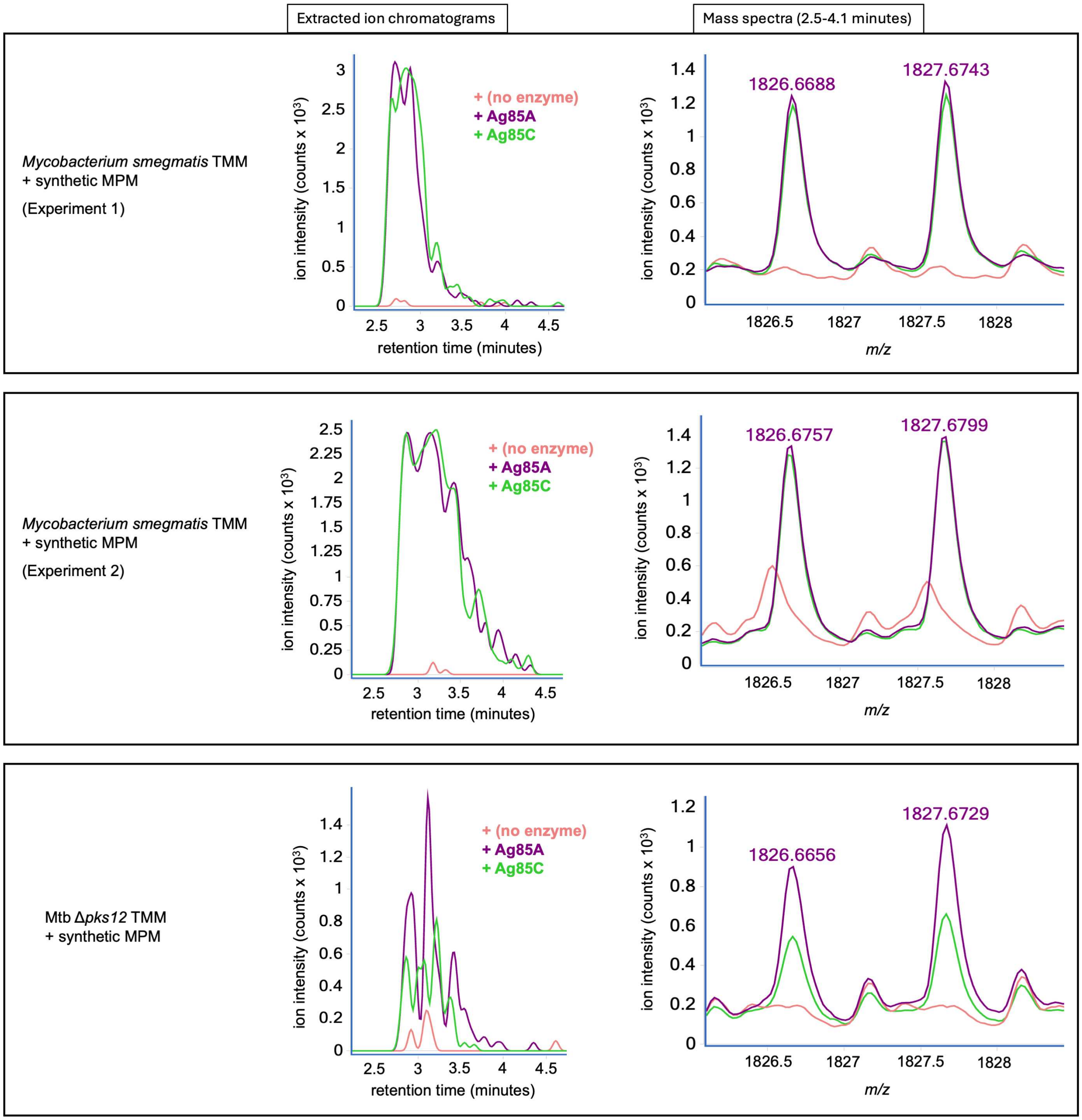

Fig. S9. Ag85 enzymes synthesize MPMMM across TMM preparations and independent experiments. HPLC-ESI-MS chromatograms (left) of the monoisotopic ion of MPMMM @ 1826 *m/z*, for products of *in vitro* reactions with or without Ag85 enzyme. Mass spectra (right) show each monoisotopic [M-H]- and the second isotope (single-^{13}C-containing) peak.
